# Aquaporin 5 Plays a Role in Estrogen-Induced Ectopic Implantation of Endometrial Stromal Cells in Endometriosis

**DOI:** 10.1371/journal.pone.0145290

**Published:** 2015-12-17

**Authors:** Xiu Xiu Jiang, Xiang Wei Fei, Li Zhao, Xiao Lei Ye, Liao Bin Xin, Yang Qu, Kai Hong Xu, Rui Jin Wu, Jun Lin

**Affiliations:** 1 Department of Gynecology, Women’s Hospital, School of Medicine, Zhejiang University, Hangzhou City, Zhejiang Province, China, 310006; 2 Department of Laboratory, Women’s Hospital, School of Medicine, Zhejiang University, Hangzhou City, Zhejiang Province, China, 310006; 3 Department of Laboratory, School of Medicine, Ningbo University, Ningbo City, Zhejiang Province, China, 315000; 4 Department of Gynecology, Sir Run Run Shaw Hospital, School of Medicine, Zhejiang University, Hangzhou City, Zhejiang Province, China, 310006; University of Quebec at Trois-Rivieres, CANADA

## Abstract

Aquaporin 5 (AQP5) participates in the migration of endometrial cells. Elucidation of the molecular mechanisms associated with AQP5-mediated, migration of endometrial cells may contribute to a better understanding of endometriosis. Our objectives included identifying the estrogen-response element (ERE) in the promoter region of the *AQP*5 gene, and, investigating the effects of AQP5 on ectopic implantation of endometrial cells. Luciferase reporter assays and electrophoretic mobility shift assay (EMSA) identified the ERE-like motif in the promoter region of the *AQP5* gene. After blocking and up-regulating estradiol (E_2_) levels, we analysed the expression of AQP5 in endometrial stromal (ES) cells. After blocking E_2_ /or phosphatidylinositol 3 kinase(PI3K), we analysed the role of AQP5 in signaling pathways. We constructed an *AQP*5, shRNA, lentiviral vector to knock out the *AQP5* gene in ES cells. After knock-out of the *AQP5* gene, we studied the role of AQP5 in cell invasion, proliferation, and the formation of ectopic endometrial implants in female mice. We identified an estrogen-response element in the promoter region of the *AQP5* gene. Estradiol (E_2_) increased AQP5 expression in a dose-dependent fashion, that was blocked by ICI182,780(an estrogen receptor inhibitor). E_2_ activated PI3K /protein kinase B(AKT) pathway (PI3K/AKT), that, in turn, increased AQP5 expression. LY294002(PI3K inhibitor) attenuated estrogen-enhanced, AQP5 expression. Knock-out of the *AQP5* gene with *AQP5* shRNA lentiviral vector significantly inhibited E_2_-enhanced invasion, proliferation of ES cells and formation of ectopic implants. Estrogen induces AQP5 expression by activating ERE in the promoter region of the *AQP5*gene, activates the PI3K/AKT pathway, and, promotes endometrial cell invasion and proliferation. These results provide new insights into some of the mechanisms that may underpin the development of deposits of ectopic endometrium.

## Introduction

Endometriosis is an estrogen-dependent, benign disease characterized by implantation and proliferation of endometrial cells on the peritoneal surfaces of the female pelvis. Whilst the retrograde menstruation theory is widely accepted as an explanation of the pathogenesis of endometriosis, it is not clear how endometrial cells implant and proliferate in ectopic sites. Aquaporins (AQPs) facilitate cell migration, invasion, and proliferation in endometrial cancer cells [1]. Aquaporin water channel proteins are abundant in a variety of cells and play a role in transcellular and transepithelial water movement [2–4]. Thirteen members of the AQP family, AQP0-AQP12, have been identified in many mammalian cells [5]. The AQP family consists of two subsets corresponding to AQPs, aqua-glyceroporins, and super-aquaporins. AQPs selectively transport water and consist of AQP0, AQP1, AQP2, AQP4, AQP5, AQP6, and AQP8. AQP5 was originally found in mammalian salivary glands [6–7]. AQP1 expression is dependent on progesterone [8]; AQP2 is regulated by estrogen [9]. Our previous studies demonstrated that AQP5 is expressed in in-situ and ectopic, endometrial glandular cells in endometriosis [10]. AQP5 is mainly located in the stromal and glandular epithelial cells of the endometrium. The expression of human endometrial AQP5 is menstrual cycle-dependent. High levels of AQP5 are found at the proliferative and mid-secretory phases, suggesting that estrogen may regulate the expression of AQP5 in endometrial glandular cells. We observed that expression of AQP5 in eutopic endometrial cells was stronger than ectopic endometrial cells. This finding suggests that eutopic endometrial cells may have stronger migration activity than ectopic endometrial cells in women with endometriosis[11–12].

Much evidence suggests that AQP5 can facilitate cell migration, proliferation and carcinogenesis in tumor development [13–17]. Our previous studies demonstrated that over-expression of AQP5 can facilitate Ishikawa (IK) cell migration (an endometrial carcinoma cell line). Knockdown of AQP5 expression in IK cells reduces cell migration [18]. We know that endometriosis cells tend to have invasion ability similar to tumor cells and that the regulatory signaling pathways are similar. We hypothesized that AQP5 may play a role in E_2_-induced formation of ectopic endometrial implants. AQP5 facilitated glandular epithelium cell migration and invasion in an endometriosis model in mice [19].

In the present study, we examined the expression of AQP5 in human, eutopic, endometrial stromal cells in the presence of 17β-estradiol (E_2_), the estrogen-response element (ERE) in the promoter region of the *AQP*5 gene, and, investigated the effects and mechanisms of AQP5 in E_2_-induced signaling pathways, stromal cell proliferation, and, formation of ectopic implants. These experiments may contribute to a better understanding of the molecular mechanisms of endometriosis.

## Materials and Methods

### Patients and tissues

Patients and eutopic endometrial tissues were collected from women attending Zhejiang Women’s Hospital for surgical treatment. Eutopic endometrial tissues were collected from 20 patients (n = 22; median age 39, range 37–44 years) with a histological confirmation of endometriosis. All 20 patients were in the follicular phase. They were confirmed by endocrine and postoperative endometrial pathologic confirmation. No patient received hormone treatment in the previous 3 months. Ethical approval of this project was granted by the Ethics Committee of Women’s Hospital, School of Medicine, Zhejiang University(**S1 File**). Written informed consent was obtained from each subject before tissue collection.

### Cells and cell culture

Eutopic endometrial tissue was rinsed in Hank’s balanced salt solution (HBSS) to remove blood and debris. The tissue digest was passed over a stacked sterile wire sieve assembly with number 100 wire cloth sieve, followed by a number 400 wire cloth sieve. The epithelial glands were retained in the number 100 and number 400 sieves, whereas the stromal cells passed through the receptacle below. Cells were cultured in Dulbecco’s Modified Eagle’s Medium and 10% fetal bovine serum (DMEM/F12, Gibco) in a humidified incubator at 37°C in an incubator with 5% CO_2_. Stromal cells were refreshed after 72 hours from the first seeding. When cells covered the bottom of the bottle, we added 0.25% trypsin enzyme (Gibco). After three cell divisions, a large number of amplifications were frozen and used. The purity of the epithelial and stromal components was assessed by cell immunohistochemical staining of cytokeratin19 (BD Pharmingen, 550953) and vimentin (BD Pharmingen. 562338) for epithelial and stromal cells, respectively, and reassessed by flow cytometry.

### Construction, identification, packaging of *AQP*5 shRNA lentiviral vectors and infection of ES cells with the lentivirus

Construction of ESAQP5-shRNA recombinant lentiviral vectors was performed as described previously [20]. Briefly, before transfection, 5×10^3^ HEK 293T cells (human embryonic kidney cells expressing the large simian virus 40 T-antigen) were seeded into each well. To identify effective shRNA targets in the *AQP*5 cDNA coding sequence (GenBank accession number: NM_001651.3), three pairs of cDNA oligonucleotides targeting the human *AQP*5 mRNA were designed using online software (http://www.sirnawizard.com/scrambled.php). One negative control shRNA containing a scrambled sequence was also designed. The three RNAi candidate target sequences and the negative control are shown in Table 1. Sense and antisense primers contained the sense siRNA sequence, and *Age* I and *Eco*R I were used to connect with the vector pLKO-GFP-TRC (Addgene). The primers used for identifying recombinant plasmids were shRNA3 (see in Table 1). ES cells were cultured in high glucose DMEM with 10% fetal bovine serum. Four types of recombinant viral supernatant and control viral supernatant were added and filtered by a 4.5-μm filter. ES cells were detected by green fluorescence after 24 h of infection. Purimycin was applied for two months and resulted in all purely expressed GFP ES cells.

**Table 1:** Three RNAi candidate target and scramble shRNA sequences.

Name	Sequence
**shAQP5-1-F**	5’-CCG GAC GCG CTC AAC AAC AAC ACA ACT CGA GTT GTG TTG TTG TTG AGC GCG TTT TTT G-3’
**shAQP5-1-R**	5’-AAT TCA AAA AAC GCG CTC AAC AAC AAC ACA ACT CGA GTT GTG TTG TTG TTG AGC GCG T-3’
**shAQP5-2-F**	5’-CCG GTG CGG TGG TCA TGA ATC GGT TCT CGA GAA CCG ATT CAT GAC CAC CGC ATT TTT G-3’
**shAQP5-2-R**	5’-AAT TCA AAA ATG CGG TGG TCA TGA ATC GGT TCT CGA GAA CCG ATT CAT GAC CAC CGC A-3’
**shAQP5-3-F**	5’-CCG GCC ATC ATC AAA GGC ACG TAT GCT CGA GCA TAC GTG CCT TTG ATG ATG GTT TTT G-3’
**shAQP5-3-R**	5’-AAT TCA AAA ACC ATC ATC AAA GGC ACG TAT GCT CGA GCA TAC GTG CCT TTG ATG ATG G-3’
**Scrambled shRNA-F**	5’-CCG GGC AAG CCC ACG CAA ATA GTT TCT CGA GAA ACT ATT TGC GTG GGC TTG CTT TTT G-3’
**Scrambled shRNA-R**	5’-AAT TCA AAA AGC AAG CCC ACG CAA ATA GTT TCT CGA GAA ACT ATT TGC GTG GGC TTG C-3’

### Electrophoretic mobility shift assay

The promoter sequence of the *AQP*5 gene was analyzed by the ENSEMBLE program to find the putative estrogen-response elements (ERE). Nuclear extracts were isolated from untreated ES cells or ES treated with 10^−8^ M E_2_ for 24 h using NE-PER Nuclear and Cytoplasmic Extraction Reagents (Thermo) according to the manufacturer’s instructions. The concentration of nuclear proteins was determined with the BCA Protein Assay Kit (Thermo), and the lysates were stored until use at -80°C. The synthetic oligonucleotides (sense and anti-sense) derived from the mouse AQP-5 promoter were synthesized by Sangon (Shanghai). Double-stranded DNA probes were 3’ end-labeled with biotin using a Biotin3’End DNA Labeling Kit (Thermo). The sense sequence of the oligonucleotide containing the putative ERE site (underlined) of the human AQP-5 promoter was 5‘-gacgacggagagcggcgggccc-3’. The sense sequence of the mutated ERE oligonucleotide was 5‘-gacgacttagatcttcgggccc-3’ (mutated base pairs in bold). EMSA was performed using a LightShift Chemiluminescent EMSA Kit (Thermo). Three micrograms of nuclear extract with protease inhibitors (Roche) and 20 fmol of biotin-labeled-ERE oligonucleotide were used for each binding reaction. A 100-fold excess of unlabeled oligonucleotide competitors was used for the competition experiments. For the super-shift reaction, 1 μg of a rabbit polyclonal IgG against the ERa (MC-20) (Santa Cruz Biotech, sc-542), ERb (H-150) (Santa Cruz Biotech, sc-8974) or normal mouse IgG (Millipore, 12-371B) was used. The reaction mixture was then loaded onto a 6% or 4% (for super-shift separation) native polyacrylamide gel, and electrophoresis was run for 1.5 h at 200 V in 0.5×TBE buffer. The protein-DNA complexes were transferred to the Biodyne B Nylon Membrane (Pierce) and UV cross-linked. Detection was performed using the enhanced chemiluminescence method included in the kit. The images were taken using the FluorChem FC2 Multi-imager II (Alpha Innotech).

### Plasmid construction and luciferase reporter assay

According to the EMSA results, a highly conserved motif was found upstream of AQP 5 (between -520 and -199 bp). Three DNA fragments of human AQP5 promoter were amplified from human endometrial DNA using the primer sequences listed in Table 2, The amplicon was then digested by Xba I and EcoRI and was subcloned between the corresponding sites in pGL3-basic to construct the AQP5 promoter luciferase reporter systems, AQP5-promoter (-0.6 kb), AQP5-promoter (-1.2 kb), and AQP5-promoter (-2.4 kb). ES cells were transfected in 24-wells multiwall and treated with estradiol for 48 h. Each sample was done in six reduplicates. Transcriptional assay was performed in dark room by adding 150 μl of phenol red-free DMEM, 150 μl of LucLite and incubating 10 min at R.T. 100 μl of each sample was transferred into a 96-well multiwall and luminescence evaluated using Wallac 1450MicroBeta TriLux (Perkin Elmer, Waltham, MA, USA). Renilla luciferase(from Renilla Reniformis) coded by pRL-TK, was then assayed in the same samples by adding 20 μl of RenLite for 30 min at R.T. and renillaluciferase activity measured using Wallac 1450 MicroBeta.

**Table 2:** Three DNA fragments of human *AQP5* promoters.

Name	Primer	Product Size (bp)
**AQP5 Promotor -0.6kb-F**	ggaggaaaaggaggagct	600
**AQP5 Promotor -0.6kb-R**	ggtggccgcgggggcccgg	
**AQP5 Promotor -1.2kb-F**	caaaaagagaaatagggttttg	1200
**AQP5 Promotor -1.2kb-R**	ggtggccgcgggggcccgg	
**AQP5 Promotor -2.4kb-F**	agtcccaggcccagaagaaaat	2400
**AQP5 Promotor -2.4kb-R**	ggtggccgcgggggcccgg	

### Western blot analysis

To determine the AQP5 and pAKT/AKT in response to ICI182,780 treatments, a second cell line were pretreated with ICI182,780 for 2h before treatment with E_2_. Recombinant cells were lysed in RIPA buffer to collect total protein (Beijing Solarbio Science & Technology Co., Ltd.,Beijing, China). After incubation on ice for 10 min, the cell lysates were precleared by centrifugation at 140,00 rpm for 15min. The protein concentration was determined using the BCA assay (Nanjing Keygen Biotech Co., Ltd.). Forty micrograms of total protein was loaded into each well and resolved by electrophoresis in a 12% SDS–PAGE gel. The proteins were electro-transferred to polyvinylidene fluoride (PVDF) membrane. After incubation with 5% of Non-fat milk for 1h at room temperate, PVDF membrane was exposed to different first antibodys at 4°C overnight, including mouse anti-AQP5 antibody (1:1000), mouse anti-AKT antibody (1:250), mouse anti-pAKT antibody (1:1000), mouse anti-pJNK antibody(1:1000), mouse anti-pERK1/2 antibody(1:1000), mouse anti-p-p38 antibody(1:1000), mouse β-tubulin monoclonal antibody (1:3000) and mouse β-actin monoclonal antibody (1:3000). After washing with TBS-T for 3 times, membrane was incubated with horseradish peroxidase-linked goat antimouse IgG antibody (1:3000) for 1 h at room temperature, and visualized with ECL detection reagent (Pierce, Thermo), bands on PVDF membrane were quantified with Image J software. All of the above antibodies were bought from Abcam.

### Effect of *AQP*5-shRNA on the growth of ES cells

To determine the growth rate of cells in response to lentiviral treatments, 5 × 10^3^ cells/well were plated in 200 μl of DMEM/F12 medium. After 24 h, the cells were cultured in DMEM/F12 medium supplemented with 10^−7^ M E_2_ and/or *AQP5-shRNA* lentivirus to assess cell proliferation using the 3-[4 5-dimethylthiazol-2-yl]-2,5-diphenyltetrazolium bromide (MTT) assay (Sigma). The A570 was measured with an enzyme-labeling instrument (BioTek, VT, USA). The alamarBlue assay was applied to quantify cell proliferation.

### Invasion assay

A Boyden chamber containing a matrigel diluted at 1:4 was used in the cell invasion experiments as the manufacturer recommended. ES cells (4 × 10^5^/ml) cultured with scrambled *shRNA*/or *AQP5shRNA* were loaded in the upper chamber. The lower chamber contained culture medium with 10^−7^ M E_2_ as a chemoattractant. Cells were incubated for an additional 6 h at 37 C in 5% CO_2_. Culture was removed from the plate. The migrated cells remaining on the bottom surface were stained with crystal violet for 30 min. Cells were photographed and counted with Image J software.

### Local injections for the mouse endometriosis model

This study was carried out in strict accordance with the recommendations in the Guide for the Care and Use of Laboratory Animals of the National Institutes of Health. The protocol was approved by the Committee on the Ethics of Animal Experiments of Zhejiang University (Permit Number: 11–3155). Twenty SCID female mice (4 weeks old, weight 70–90 g, animal license SCXK (shu) 2011–0003, mouse feeding environment conformed to the requirements of the animal ethics committee) were divided into two groups: 10 in the scrambled shRNA group and 10 in the ES-*AQP*5shRNA group. To improve the formation rate of ectopic lesions, SCID mice were given 10^−7^ M E_2_ subcutaneously. Mice were briefly anesthetized with 2.5% isoflurane to facilitate the injection of drugs into the ES cells or ES-*AQP*5shRNA cells located in the abdominal cavity (0.5 ml cell suspension). The density of injected cells was 1×10^7^/mL. Injected cells were tracked by GFP. The injection site was previously shaved and scrubbed with alcohol. Mice were killed 15 days after the injection. Nodules were stripped and confirmed under a fluorescence microscope. Ectopic implants were proved by pathology.

### Statistical analyses

All data were normally distributed and are expressed as the mean±SEM. The independent-samples t test was used to evaluate the statistical significance of the difference between two groups. SPSS version 16.0 (SPSS Inc., Chicago, IL) was used for the statistical analysis. A P value less than 0.05 was considered statistically significant.

## Results

### Construction of primary ES cells and ES*AQP*5-shRNA recombinant lentiviral vectors

**Fig 1:**
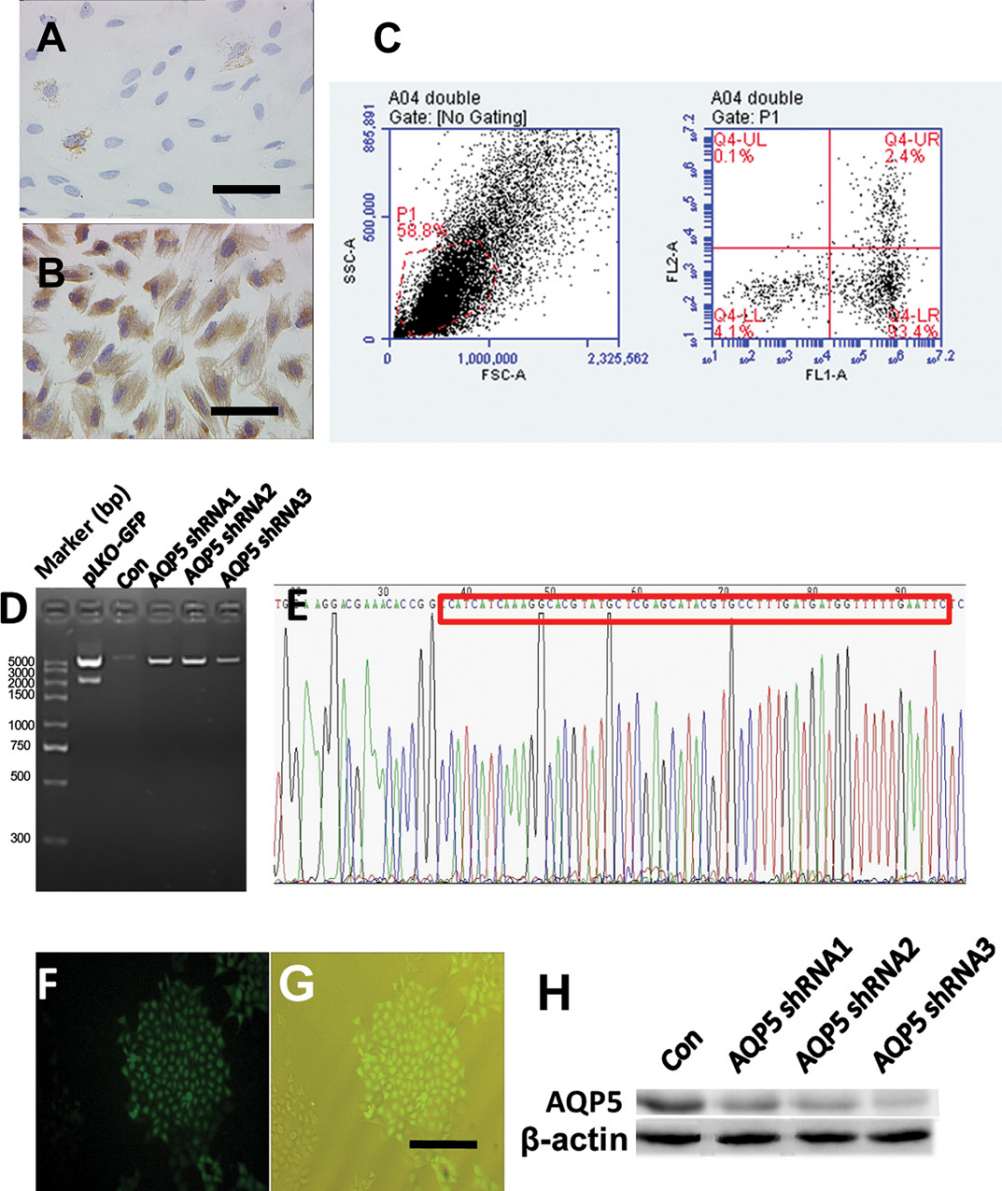
Construction of primary ES cells and ESAQP5-shRNA recombinant lentiviral vectors. After three cell divisions, the ES density was approximately 99%. Glandular epithelial cells were stained by cytokeratin 19 by immunocytochemistry (A, original magnification, ×400). Stromal cells were stained by Vimentin (B, original magnification, ×400). The bottom right corner of the black horizontal line is 10 μm. Flow cytometry showed that both CK19 and Vimentin positive cells accounted for 2.4% of the total, single CK19 positive accounted for 0.1%, single Vimentin positive accounted for 93.4%, and both CK19 and Vimentin negative accounted for 4.1% (C). The shRNA insert is 2155 bp, and the empty plasmid is 2155 bp (D). The sequencing showed that the cloned AQP5shRNA sequence was inserted correctly without mutation (E). pLKO-GFP-TRC–AQP5shRNA was transformed into ES cells and confirmed by GFP under the fluorescence field of view (F) and under the bright field view (G). The recombinant plasmids were selected (H). Con means the negative control of scrambled shRNA that inserted into pLKO-GFP after enzyme digestion.

Adherent growth of the primary ES cells presented a purity of 99.9% (Fig 1A, 1B and 1C). *AQP*5shRNA PCR-amplified products were linked into the pLKO-GFP-TRC vector. The recombinant plasmid cut with the same enzyme produced bands of 7872 bp and 2155 bp (Fig 1D). The sequencing showed that the cloned *AQP*5shRNA sequence was inserted correctly without mutation (Fig 1E). pLKO-GFP-TRC–*AQP*5shRNA was transformed into ES cells and confirmed by GFP (Fig 1F and 1G). Weak AQP5 expression on panel AQP5shRNA3. The recombinant plasmids(shRNA3) were selected by puromycin and identified by western blot (Fig 1H).

### Identification of an ERE in the 5’ flanking region of *AQP*5

**Fig 2:**
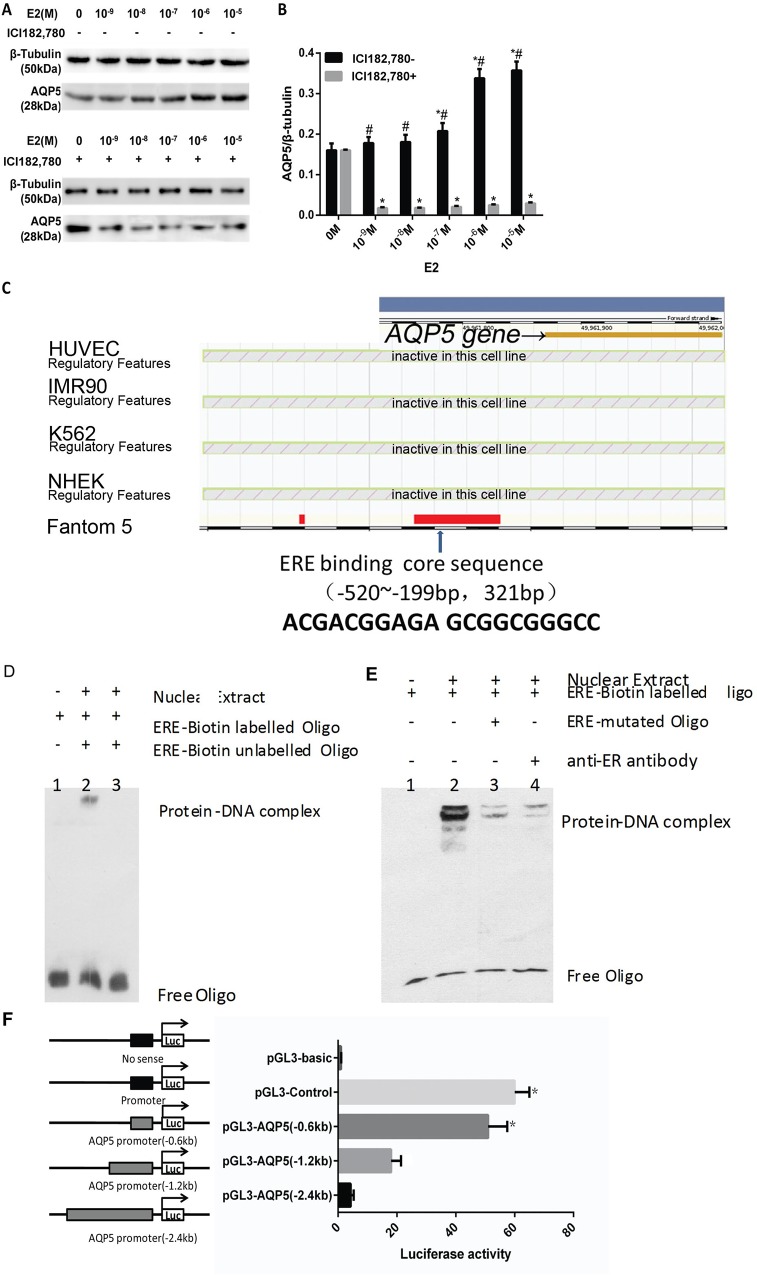
Effects of E_2_ on AQP5 expression in ES cells and AQP5 is a target of ERα. E_2_ effect on AQP5 protein expression (A and B). # P < 0.01 compared with values from control;* P < 0.01, ICI182,780+ group compared with corresponding values in the ICI182,780- group. Putative ERE sequence in the promoter of the *AQP5* gene are indicated (C). Binding of nuclear proteins to oligonucleotide contain the putative *AQP5* promoter ERE-site (D, E). Nuclear extracts prepared from ES cells treated with 10^−8^ M E_2_ were incubated with the biotin-labeled ERE oligonucleotide probe either in the absence (lane 2) or presence of a 100-fold excess of unlabeled ERE oligonucleotide (lane 3). As a control, a reaction without the nuclear extract was performed (lane 1) (D). Binding of ERα to a putative ERE in *AQP5* promoter (E). Nuclear extracts prepared from ES cells treated with 10^−8^ M E_2_ were incubated with the biotin-labeled ERE oligonucleotide probe either in the absence (lanes 2) or presence of ERE-mutated Oligo (lane 3) or anti-ERα(lane 4) polyclonal antibodies in a reaction mixture. As a control, a reaction without the nuclear extract was performed (lane 1). The transcriptional initiation site is indicated by the arrow (F). The promoter is shown as a thin line. Exons and introns are indicated by solid black boxes and open boxes, respectively. The positive control (pGL3-basic) contained three canonical ERE elements. Three constructs contained the 5’ flanking region of *AQP5* (pGL3-AQP5 0.6 kb, pGL3-AQP5 1.2 kb, and pGL3-AQP5 2.4 kb) and a negative control (pGL3-control). The response of 5’ flanking regions of *AQP5* to estrogen stimulation. Luciferase reporter constructs were transfected into HEK293 cells with an ERα expression vector and a control plasmid and then examined in the presence or absence of 10^−7^ M estrogen. Luciferase intensities detected in the presence of estrogen were divided by those in the absence of estrogen and the relative differences calculated. * P < 0.01 compared with corresponding control.

E_2_ increased AQP5 expression in a dose-dependent fashion at 10^−9^ to 10^−5^ M in ES cells (Fig 2A). The up-regulated expression of AQP5 protein was partially blocked by pretreatment of cells with the estrogen receptor inhibitor ICI182,780 at 10^−9^ to 10^−5^ M (Fig 2B), indicating that estrogen and other hormone receptor were involved.

To determine whether the *AQP*5 gene is a target of ER, we used the ENSEMBLE program to investigate the *AQP5* gene by ERαin the upstream region of the *AQP5* gene. A highly conserved motif was found upstream of AQP5 (between -520 and -199 bp, Fig 2C). To confirm the binding of ERα to the putative ERE, we performed a conventional EMSA with fragments from the 5’ flanking and coding regions of AQP5 (Fig 2D, Table 2). Only fragments containing the putative ERE and its adjacent sequences could be precipitated following administration of estrogen (Fig 2E). To determine whether the putative ERE plays a functional role in estrogen-dependent transcriptional activation (Fig 2F), we constructed three luciferase reporter plasmids (named pGL3-AQP5 0.6 kb, pGL3-AQP5 1.2 kb and pGL3-AQP5 2.4 kb) containing the 5’ flanking point of AQP5 (0.6 kb, 1.2 kb and 2.4 kb, upstream of the ATG translation initiation site). Only the positive control and the construct containing putative ERE (Pgl3-AQP5 0.6 kb) were activated by estrogen, whereas the constructs that did not contain the putative ERE were unaffected. These results suggest that there is a functional ERE element in the 5’ flanking region of AQP5 that is directly regulated by ERα.

### Effects of E_2_ on AQP5 expression by activating the PI3K/AKT pathway

**Fig 3:**
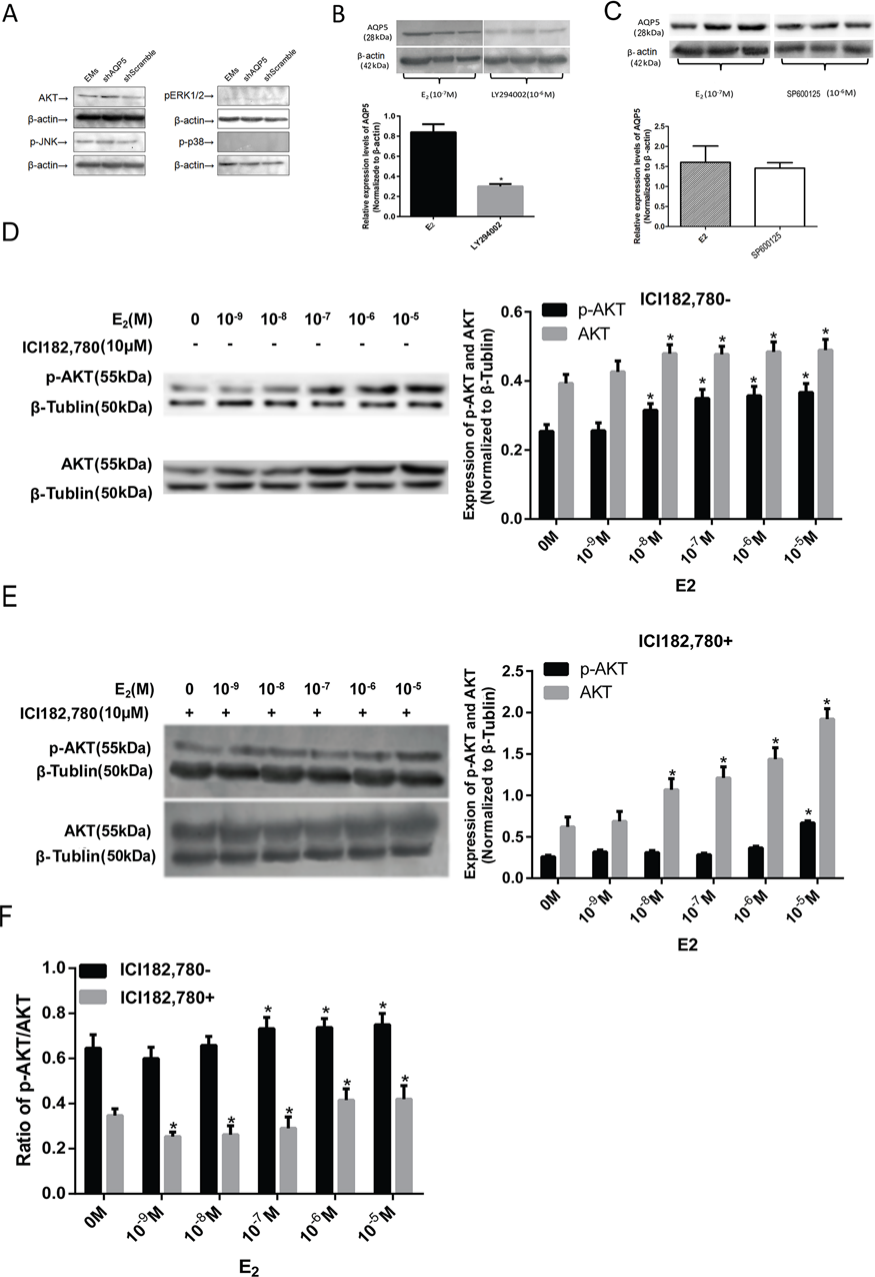
E_2_ regulates AQP5 expression by the PI3K/AKT signaling pathway. Data are from a second cell line. Related signal pathways in endometrial stromal cells after AQP5-gene silence(A):shAQP5 means knock out *AQP5* gene. shScramble means random sequence which won’t knock out target sequence. Expression levels of AQP5 protein induced by E_2_ were blocked by LY294002(B) or SP600125(C). * P < 0.01 compared with control group. The number of repeated experiments is three. E_2_ effects on AKT and pAKT expression (D,E). * P < 0.01 compared with values from control. The ICI182,780 effects on pAKT and AKT, * P < 0.01 E_2_ group compared with values from ICI182,780 (F).

To determine which signal pathway relevant with AQP5, four signal pathways were detected. After *AQP5* gene was knock out, AKT and JNK signal pathways were not influenced, but ERK and p38 pathways were not expressed in stromal cells(Fig 3A). Compared with the E_2_ group, LY294002 (a PI3K inhibitor, 10^−6^ M) partially attenuated E_2_ enhanced AQP5 expression (Fig 3B), while SP600125(a JNK inhibitor, 10^-6^M) didn’t influence AQP5 expression(Fig 3C). It implied AQP5 was the downstream of PI3K/AKT pathway. Western blot analysis showed that AKT was the upstream signal pathways in endometrial stromal cells after AQP5- gene silence. E_2_ at 10^−9^ to 10^−5^ M dose dependently increased both Akt and pAkt levels in ES cells (Fig 3D and 3E). ICI182,780 at 10^−5^ M decreased both AKT and pAKT levels at various E_2_ levels. The reduced ratio of pAKT was statistically higher than that of AKT (Fig 3F).

### Inhibition of proliferation, invasion, and ectopic implant formation of ES cells by *AQP*5 shRNA

**Fig 4:**
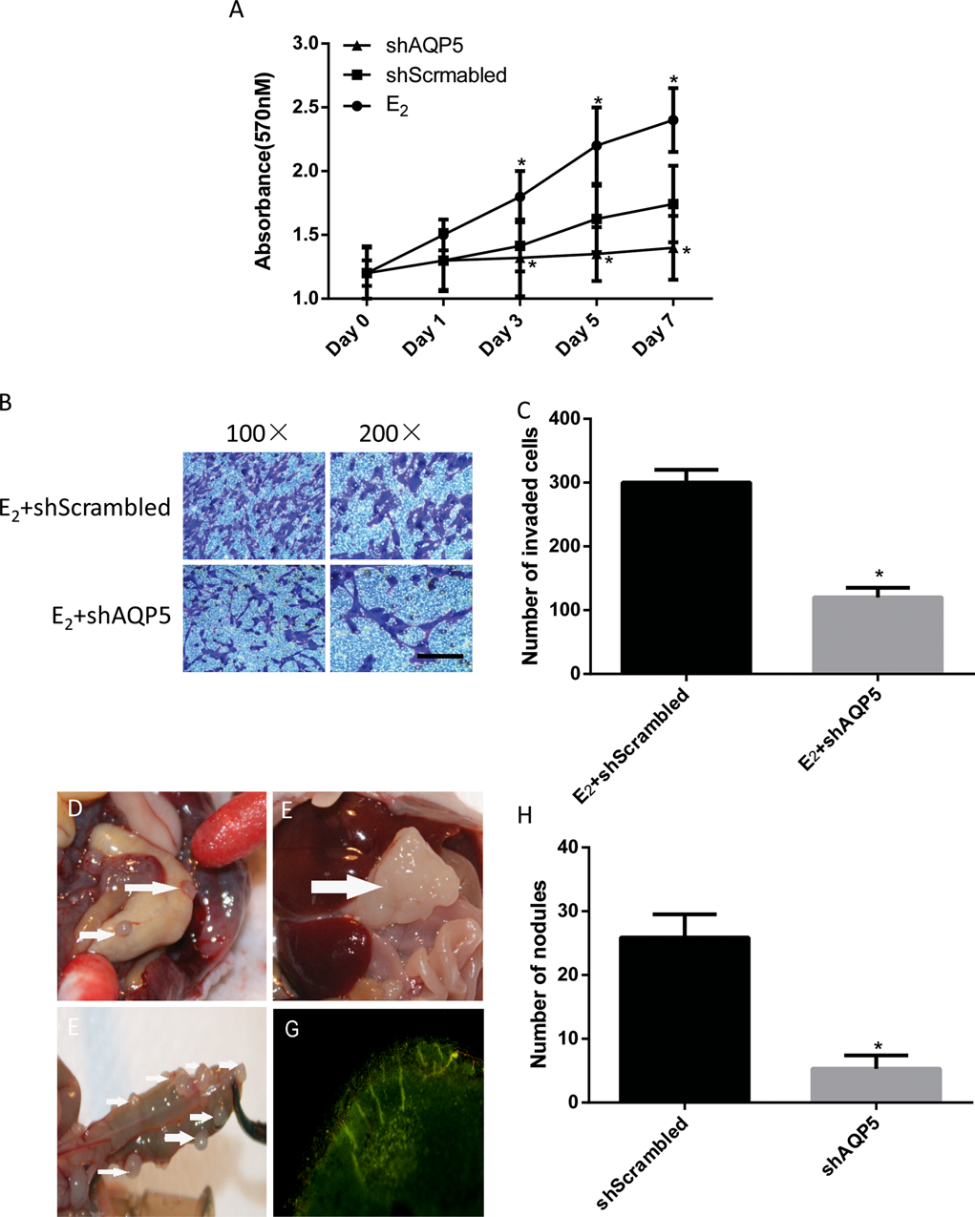
Role of AQP5 in cell proliferation, invasion and ectopic implant formation. The role of AQP5 in cell proliferation was shown by a cell growth curve (A). E_2_ (10^−7^ M) effects on cell proliferation (A) and invasion (B,C). * P<0.05 compared with scrambled. Ectopic nodules were observed in the abdominal cavity in the control group (D,G). Numbers and volumes of nodules were observed in the *AQP*5 gene knockout group (E,F). The formation of the nodules from our GFP positive cells were traced and confirmed with GFP by a fluorescence microscope(G). Compare of nodules in the control group and in the *AQP*5 shRNA group (H).

Recombinant cells with *AQP*5-shRNA not only reduced proliferation but also significantly inhibited invasion (Fig 4A–4C). Knockout of the *AQP*5 gene had a similar effect on the E_2_-enhanced ectopic implant formation of ES cells (Fig 4D–4H).

Both groups had a 100% nodule formation rate. One mouse died in each group. Gray, irregular and hard ectopic nodules scattered throughout the greater omentum, mesentery, and liver surfaces in the control group (Fig 4D). In contrast, smaller numbers and volumes of nodules were observed in the *AQP*5 gene knockout group (Fig 4E and 4F). The largest nodule in the *AQP*5 gene knockout was 1.5×1.5×2.0 cm. All of the nodules contained GFP tags confirmed by fluorescence microscopy(Fig 4G). There were significantly more nodules in the control group than in the *AQP*5 shRNA group (5.3±2.1 VS 25.9±3.6, p<0.05) (Fig 4H).

## Discussion

An understanding of the mechanisms controlling cell migration and proliferation in the endometrium may aid our understanding of endometriosis. The present study demonstrates the following: 1) Estradiol increases AQP5 expression in endometrial stromal cells in a dose-dependent fashion, and there is an ERE in the promoter of *AQP*5; 2) AQP5 plays a role in E_2_-enhanced invasion and proliferation of ES cells by activating the PI3K/AKT pathway; and 3) AQP5 mediated the formation of E_2_-enhanced ectopic implants in vivo. Variant sequences of ERE have been reported in mouse uterus [21–22]. Our luciferase reporter system and EMSA analyses demonstrated an ERE in the *AQP*5 promoter in human uterus. Our results suggest that E_2_ may regulate *AQP*5 expression via an ERE in the *AQP*5 promoter region. The E_2_ effect was not totally blocked by ICI182,780, indicating that other factors, such as progesterone, may influence AQP5 expression, as we have found that AQP5 has the highest expression during the late proliferative and early secretory phases [10].

In a previous study, we demonstrated that the expression of ectopic, endometrial AQP5 in patients with endometriosis is menstrual cycle-dependent, and, that AQP5 plays a role in endometrial cancer cell migration [19]. The findings in this study confirm that E_2_ regulates AQP5 expression in a dose-dependent fashion. We observed that AQP5 promoted the invasion and proliferation of epithelial cells in an endometriosis model [19]. AQP5-enhanced invasion and proliferation of ES cells provides further evidence of the involvement of AQP5 in the development of endometriosis. It has been reported that AQP5 may play a role by activating ERK or P38/MAPK signaling pathways [23–24]. Currently, it is not clear whether phosphorylation-signaling pathways are involved in the regulation of steroid hormones linked to ES cell invasion and proliferation. Some data from malignant tumors suggest that AQP5 over-expression is related to the PI3K phosphorylation and activation of Ras signaling pathways [25–26]. Our results show that the inhibitor of PI3K partially attenuated E_2_-enhanced AQP5 expression. The scrambled *AQP5* gene also seems to have a proliferative effect on cells. AQP5 facilitates E_2_-induced ES cell invasion and proliferation by activating the PI3K/AKT pathway. The present study found that estrogen had a growth factor-like function. Estrogen increases AQP5 protein expression and the ability of ES cells to proliferate, which was positively correlated with PI3K/AKT phosphorylation. After knockout of the *AQP*5 gene, both ES cell proliferation and invasion were suppressed. We hypothesize that E_2_ regulates AQP5 expression in a two-way fashion. On the one hand, E_2_ combined AQP5 directly, on the other hand, E_2_ regulates AQP5 by activating AKT pathway. This study is the first time that we have confirmed that a decline in AKT phosphorylation may suppress cell proliferation and invasion in an *AQP*5 knockout ES cell model, which also provides a theoretical basis for the enhanced biological effects of *AQP*5 over-expression in other types of cells, such as breast cancer and lung cancer cells [27–30].

A primary culture of eutopic endometrial stromal cells is a useful technique to study the behavioral characteristics of endometriosis cells. Interference with the target protein in a lentiviral vector is more stable and complete because it is based on the reconstruction of an HIV carrier in which target genes can be integrated into the genome, whereas cell-acquired traits will not be lost by cell passage. Therefore, by establishing a stable knockout *AQP*5 lentivirus ES cell, we can make ES AQP5 protein permanently silent in the daughter cells, change related upstream and downstream pathways permanently, and achieve a more stable platform for in vitro research. Retrograde flux of endometrial cells is a major cause of ectopic endometrial implantation in the female pelvis. By comparing two types of endometrial stromal cells in the abdominal cavity, the present study confirmed that there is decreased implantation of ectopic endometrium in the abdominal cavity with knockout of the *AQP*5 gene in eutopic endometrial stromal cells. Knockout of *AQP*5 expression and/or function may be a useful potential therapy to reduce the development of endometriosis.

In summary, our findings identified an ERE in the promoter region of *AQP5*, and that increased AQP5 protein expression by E_2_ might be a downstream signal for AKT phosphorylation signaling pathways that promote cell proliferation and invasion. We confirmed that the *AQP*5 gene may contribute to the formation of ectopic endometrial implants in vivo. These results provide new avenues for the investigation and treatment of endometriosis by inhibiting expression and function of aquaporin channels.

## Supporting Information

S1 File Institutional Review Board support for the project. Ethical approval of this project was granted by the Ethics Committee of Women’s Hospital, School of Medicine, Zhejiang University.
(PDF)
