# Supplementary material for: Aquaporin 5 Plays a Role in Estrogen-Induced Ectopic Implantation of Endometrial Stromal Cells in Endometriosis
Source: PLoS One. 2015 Dec 17;10(12):e0145290. doi: 10.1371/journal.pone.0145290 (PMC4682985; doi:10.1371/journal.pone.0145290)
Supplement: S1 File — Ethical approval of this project was granted by the Ethics Committee of Women’s Hospital, School of Medicine, Zhejiang University. (PDF) [file pone.0145290.s001.pdf]

浙江大学医学院附属妇产科医院

医学伦理委员会意见书

<table>
<tr><td>项目类型</td><td colspan="5">1、临床科研　√、临床基础科研　3、药物验证　4、医疗仪器试剂验证
5、新技术开展</td></tr>
<tr><td>项目名称</td><td colspan="3">水通道蛋白介导痛觉过敏在子宫内膜异位症慢性盆腔痛发生机制中的研究</td><td>伦理批准号</td><td>20120070</td></tr>
<tr><td>项目负责人</td><td>江秀秀</td><td>职称</td><td>副主任医师</td><td>所在科室</td><td>妇科</td></tr>
<tr><td>项目参与者</td><td colspan="5">田永红，温洁，马俊彦，詹宏，阿斯燕.努斯拉提，张晓鑫</td></tr>
<tr><td>项目实施的伦理学问题</td><td colspan="5">1、对受试者可能带来的心理、生理、生活、经济及其他损害和不利影响，防范和补救对策。
2、对受试者可能带来的近、远期潜在危险及其防范
3、其它伦理学问题（包括受试者个人、家庭、社会、人类等方面）

1.对受试者可能带来的心理、生理、生活、经济及其他损害和不利影响，防范和补救对策。
实验需要构建子宫内膜异位症动物模型。所需内膜来自子宫内膜异位症患者的在位子宫内膜 20 例。构建原代子宫内膜细胞株：选取同一个患者的在位子宫内膜，验证子宫内膜上 AQPs 的表达。
子宫内膜异位症患者行内异囊肿剔除或子宫切除术中全麻或硬膜外麻醉下刮取无生育要求患者的在位子宫内膜约 1.0g，严格无菌操作，无疼痛。对受试者不会带来的心理、生理、生活、经济及其他损害和不利影响。不会影响患者疾病的诊断和治疗。
2、对受试者可能带来的近、远期潜在危险及其防范
对受试者不会带来近、远期潜在危险。
3、其它伦理学问题（包括受试者个人、家庭、社会、人类等方面）
无其他伦理学问题</td></tr>
</table>

<table>
<tr><td>报送材料</td><td>1、知情同意书
2、
3、
4、
5、
6、
7、
8、
9、
10、
申请者签名：江秀秀</td></tr>
<tr><td>伦理委员会意见</td><td>是否符合简易程序：
1、是 ✓
2、否
审查结果：
1、同意 ✓
2、修改后同意
3、不同意
伦理委员会主任（签名）：[illegible]
2012年 5月20日</td></tr>
</table>

项目名称：水通道蛋白介导痛觉过敏在子宫内膜异位症慢性盆腔痛发生机制中的研究
